# Conserved infections and reproductive phenotypes of *Wolbachia* symbionts in Asian tortrix moths

**DOI:** 10.1111/1758-2229.13219

**Published:** 2023-12-09

**Authors:** Hiroshi Arai, Masatoshi Ueda, Tatsuya Hirano, Naoya Akizuki, Shiou‐Ruei Lin, Duong Kieu Hanh, Jaka Widada, Muhammad Saifur Rohman, Madoka Nakai, Yasuhisa Kunimi, Le Van Vang, Arman Wijonarko, Maki N. Inoue

**Affiliations:** ^1^ United Graduate School of Agricultural Science Tokyo University of Agriculture and Technology Tokyo Japan; ^2^ Crop Environment Section Tea and Beverage Research Station, Ministry of Agriculture Taoyuan City Taiwan; ^3^ College of Agriculture Can Tho University Can Tho City Vietnam; ^4^ Department of Agricultural Microbiology, Faculty of Agriculture Universitas Gadjah Mada Yogyakarta Indonesia; ^5^ Department of Plant Protection, Faculty of Agriculture Universitas Gadjah Mada Yogyakarta Indonesia

## Abstract

*Wolbachia* is a ubiquitous endosymbiotic bacterium that manipulates insect reproduction. A notable feature of *Wolbachia* is male killing (MK), whereby sons of infected females are killed during development; however, the evolutionary processes by which *Wolbachia* acquired the MK ability remain unclear. The tea tortrix moth *Homona magnanima* (Tortricidae) harbours three non‐MK *Wolbachia* strains (*w*Hm‐a, *w*Hm‐b and *w*Hm‐c) and an MK strain *w*Hm‐t. Although *w*Hm‐t and *w*Hm‐c are closely related, only *w*Hm‐t has an MK‐associated prophage region. To understand the evolutionary processes underlying the emergence of MK *w*Hm‐t, we examined *Wolbachia* infections and phenotypes in 62 tortricid species collected from 39 localities across Japan, Taiwan, Vietnam and Indonesia. PCR assays detected *w*Hm‐c relatives in 51 species and triple infection of *w*Hm‐a, *w*Hm‐b and *w*Hm‐c in 31 species. Apart from Taiwanese *H. magnanima*, no species exhibited the MK phenotype and were positive for the *w*Hm‐t‐specific prophage. While *w*Hm‐t infection was dominant in Taiwanese *H. magnanima*, *w*Hm‐a, *w*Hm‐b and *w*Hm‐c were dominant in Japanese *H. magnanima* populations. These results suggest that *w*Hm‐a, *w*Hm‐b and *w*Hm‐c strains descended from a common ancestor with repeated infection loss and that *w*Hm‐t evolved from the *w*Hm‐c acquiring MK ability in allopatric populations of *H. magnanima*.

## Graphical abstract

By analysing tortrix moths collected from Japan (JPN), Taiwan (TWN), Vietnam (VNM) and Indonesia (IDN), we reveal a conserved *Wolbachia* infection in Asian tortrix moths. Furthermore, we show distinct infection patterns of male‐killing (MK) and non‐MK *Wolbachia* strains in *Homona magnanima* populations. We conclude that the *Wolbachia* strains are descended from a common ancestor with repeated loss of infection and that the MK strain *w*Hm‐t has evolved from its closely related *w*Hm‐c strain in allopatric populations of *H. magnanima*.

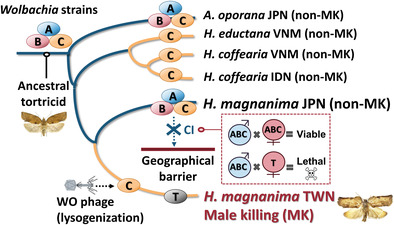

## INTRODUCTION

A maternally inherited intracellular bacterium, *Wolbachia* (*Alphaproteobacteria*), is found in at least 40% of all insect species, making it one of the most widespread endosymbionts (Rasgon et al., 2006; Stouthamer et al., 1999; Werren et al., 2008; Zug & Hammerstein, 2012). *Wolbachia* has achieved evolutionary success by manipulating host reproduction through various means that enhance endosymbiont transmission. These manipulations of the host reproduction and development include cytoplasmic incompatibility (CI), male killing (MK), parthenogenesis and feminisation, each of which seemingly affects the biological features, distribution and evolution of the host (Charlat & Merçot, 2003; Charlat et al., 2007; Jiggins, 2003; Narita et al., 2007; Rokas, 2000; Turelli & Hoffmann, 1991; Werren et al., 2008). For instance, MK directly skews the sex ratio of the host population towards females, allowing *Wolbachia* to spread the infection across the host population (Werren et al., 2008).

For decades, *Wolbachia*‐induced MK has drawn the attention of basic and applied biologists. One unresolved question is how *Wolbachia* acquired the MK ability through evolutionary processes. *Wolbachia* occasionally transmits horizontally among arthropods but is primarily transmitted from female hosts to their offspring (Duron et al., 2008; Vavre et al., 1999; Werren et al., 2008). Previous studies have suggested that sister taxa of host species harbour closely related *Wolbachia* (Baldo et al., 2006; Kageyama et al., 2004; Watanabe et al., 2011; Watanabe et al., 2012). For instance, closely related MK‐inducing *Wolbachia* strains are conserved among *Ostrinia* moths (Kageyama et al., 2004; Muro et al., 2023). In this case, an ancestral *Wolbachia* strain likely acquired MK properties and was descended from its host species. In contrast, closely related *Wolbachia* strains do not necessarily exhibit similar phenotypes (Arai et al., 2020; Metcalf et al., 2014). The phylogenetic relationship between MK *Wolbachia* strains and their host is not always concordant, suggesting that *Wolbachia* independently acquired their MK abilities multiple times (Baldo et al., 2006; Bleidorn & Gerth, 2018; Werren et al., 2008; Zhou et al., 1998). To understand the evolution of an MK *Wolbachia* strain, it is critical to determine whether MK *Wolbachia* descended from the ancestral host species or whether *Wolbachia* acquired an MK ability independently in the host.

Here, to clarify the evolutionary processes through which a *Wolbachia* strain *w*Hm‐t acquired MK abilities in the oriental tea tortrix moth *Homona magnanima* (Tortricidae, Lepidoptera), we report the comprehensive analysis of *Wolbachia* infections and phenotypes in Tortricidae, including *H. magnanima* and 61 tortricid species, collected from 39 populations across Japan, Taiwan, Vietnam and Indonesia. We previously reported that *H. magnanima* was co‐infected with three *Wolbachia* strains in several Japanese populations (Arai et al., 2019; Takamatsu et al., 2021). Among them, *w*Hm‐a exhibited no apparent effects on the host; *w*Hm‐b induced CI that impaired the development of offspring of infected males and non‐infected females, while *w*Hm‐c promoted host fecundity by increasing the pupal weight of the hosts (Arai et al., 2019; Ueda et al., 2023). In contrast, Taiwanese *H. magnanima* harbours an MK strain, *w*Hm‐t, which is closely related to the non‐MK strain *w*Hm‐c (Arai et al., 2020). More recently, we identified an MK‐associated prophage region that is present in *w*Hm‐t but absent in *w*Hm‐c (Arai et al., 2023). In the current study, we report (i) the conserved infections of *Wolbachia w*Hm‐a, *w*Hm‐b and *w*Hm‐c relatives among Asian tortricids and (ii) the restricted distribution of the MK *w*Hm‐t in Taiwanese *H. magnanima*. Our investigations provide insights into the evolutionary processes by which a non‐MK *Wolbachia* acquired the MK ability and how *Wolbachia* strains have persisted in their host populations.

## EXPERIMENTAL PROCEDURES

### Insects

*H. magnanima* and other tortricids were collected from damaged tea leaves by hand or using UV light and sex pheromones (Sumitomo Chemical Co., Ltd., Tokyo, Japan) in Japan, Vietnam and Indonesia. Taiwanese *H. magnanima* were previously obtained by Arai et al. (2020). The species name of the collected tortricids was determined based on morphological characters described in Kishida (2011), Tortricid.net (http://www.tortricidae.com/identification.asp), and BOLD database (https://www.boldsystems.org/index.php/TaxBrowser_Home). Samples were stocked in ethanol at −35°C until analysis.

Larvae were reared individually until eclosion with an artificial diet INSECTA LF (Nosan Co. Ltd., Yokohama, Japan) at room temperature (20–26°C) under 14–16 L:8–10D conditions. Adults were mated in a plastic bag (20 × 30 cm), and wax paper was placed to obtain egg masses. A collected egg mass was placed on sliced INSECTA LF in a plastic container (20 × 30 × 10 cm) for mass rearing. The sex ratio ([number of males/number of females] among enclosed adults) was determined.

### DNA extraction, PCR and sanger sequencing

DNA was extracted as described by Arai et al. (2019) or with DNA zol (Molecular Research Center, Inc., OH, USA) following the manufacturer's protocol. *Wolbachia wsp* gene and conserved Lepidopteran *COI* gene were amplified with Emerald Amp Max Master Mix (TaKaRa Bio. Co. Ltd., Shiga, Japan) with specific primer sets, *COI*: LepF (5′‐ATTCAACCAATCATAAAGATATTGG‐3′) and LepR (5′‐TAAACTTCTGGATGTCCAAAAAATCA‐3′) (Hajibabaei et al., 2006); *wsp*: wspF81 (5′‐TGGTCCAATAAGTGATGAAGAAAC‐3′) and wspR691 (5′‐TGGAGTAGCGTTTAATTTTT‐3′) (Zhou et al., 1998). To distinguish *Wolbachia* genotypes, primers for *w*Hm‐a: F321 (5′‐CCTAAACAAAAATAATGTTACAG‐3′) and R565 (5′‐TTTGATCATTCACAGCGT‐3′); *w*Hm‐b: F176 (5′ GGTGCTAAAAAGAAGACTGCGG‐3′) and R667 (5′‐CCCCCTTGTCTTTGCTTGC‐3′); *w*Hm‐c: F188 (5′‐CATATAAATCAGGTAAGGACAAC‐3′) and R603 (5′‐CACCAGCTTTTGCTTGATA‐3′), were employed. PCR conditions were as follows: 2 min at 94°C, 35 cycles of 30 s at 94°C, 30 s at annealing temperature (50°C for *w*Hm‐a, 55°C for *w*Hm‐c and *COI*, 60°C for *w*Hm‐b and universal *wsp*), 30 s at 72°C and 3 min at 72°C. The presence or absence of the *w*Hm‐t‐specific prophage region in field‐collected tortricid species was assessed using PCR by amplifying a *Hm‐oscar* gene with the following primers: WHMT_00358_f (5′‐ATGATTGAAGATAGAAATGTTCCTTTATCC‐3′) and WHMT_00358_r (5′‐CTACCTACCGCCTTTACCTTTGCTA‐3′) with the Emerald Amp Max Master Mix (TaKaRa) at 94°C for 3 min, 35 cycles of 94°C for 30 s, 62°C for 30 s, 72°C for 3 min and final extension at 72°C for 7 min.

PCR products were purified using the Qiaquick PCR purification kit (Qiagen, Hilden, Germany) and sequenced as described by Arai et al. (2019). Purified *wsp* amplicons of *Wolbachia* in *Homona* species were ligated with the pGEM‐T easy vector (Promega, Madison, WI, USA) and transfected into JM109 competent cells. Plasmid DNAs extracted from eight JM109 clones using the Pure Yield Plasmid Miniprep System (Promega, Madison, WI, USA) were sequenced as described by Arai et al. (2019).

### *Constructions of the mitochondrial genome of* H. magnanima

The mitochondrial genome of a tortricid, *Choristoneura fumiferana* (accession number: NC_037395.1), retrieved from the NCBI database, was used as a reference to identify mitochondrial reads from sequence reads of *H. magnanima* (DRA013595, BioProject: PRJDB13119) using BLASTn search. A complete mitochondrial genome was constructed through contig alignments and read mappings using minimap2 (Li, 2018), followed by manual cyclisation. Resequencing data of *H. magnanima* obtained from Japanese and Taiwanese populations (DRA013650, BioProject: PRJDB13119) were mapped to the mitochondrial genome of *H. magnanima* using minimap2, and consensus sequences were obtained using SAMtools (Li et al., 2009).

### Phylogenetic analysis

Newly sequenced *wsp* fragments (*Wolbachia*) and mitochondrial genomes (*H. magnanima*) and COI fragments (tortricids) were aligned using ClustalW (Thompson et al., 1994). *Wolbachia wsp* sequences of Baldo et al. (2006) were retrieved to construct a *Wolbachia* phylogenetic tree. For phylogenetic analysis, the maximum likelihood method with bootstrap re‐sampling of 1000 replications was performed in MEGA 7 (Kumar et al., 2016). Haplotype network analysis was conducted on mitochondrial COI sequences of *H. magnanima* using DnaSP6 (Rozas et al., 2017) and Network (Bandelt et al., 1999).

### Statistical analysis

To examine whether *Wolbachia* infections correlate with host sex, GLMM was performed using the lme4 package (Bates et al., 2015) in R 4.0.0, assuming binomial error based on the presence/absence of the respective endosymbionts within an individual *H. magnanima* as shown by Takamatsu et al. (2021). In statistical modelling, we considered the infection status (i.e., *Wolbachia* presence or absence) as a fixed factor. Based on the GLMM, an analysis of variance (ANOVA) was conducted to evaluate the significance of the individual model terms.

## RESULTS

### Asian tortricids generally harboured *w*Hm‐a, *w*Hm‐b and *w*Hm‐c relatives but lacked a female‐biased sex ratio

**FIGURE 1:**
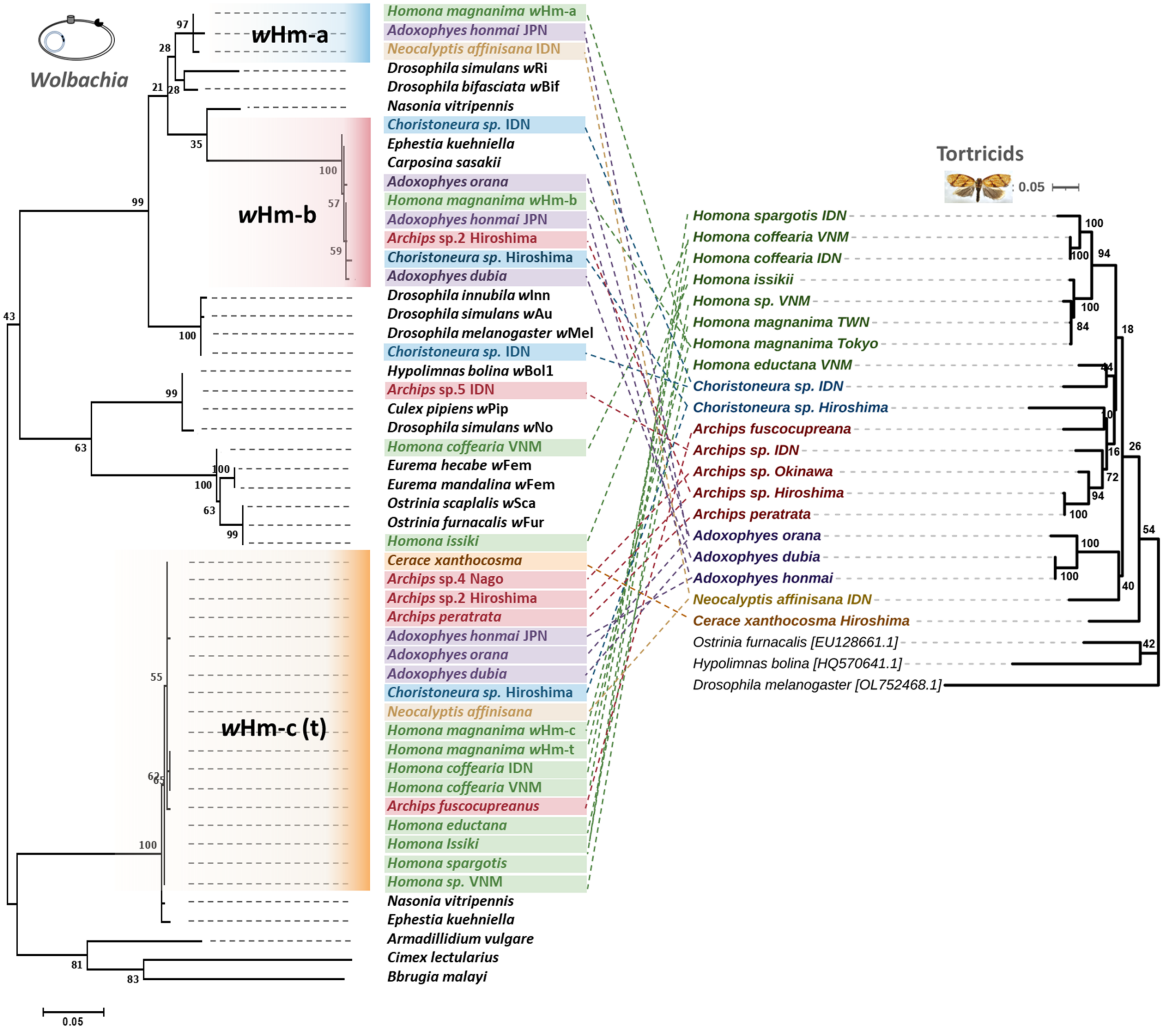
Phylogeny of *Wolbachia* strains identified from tortricids. The *wsp* sequences of *Wolbachia* strains in tortricids were aligned with those of other *Wolbachia* strains reported by Baldo et al. (2006). The mitochondrial *COI* sequences of tortricids were also shown. The phylogenetic tree was constructed using the maximum likelihood method based on the Tamura–Nei model with 1000 bootstrap replicates.

**FIGURE 2:**
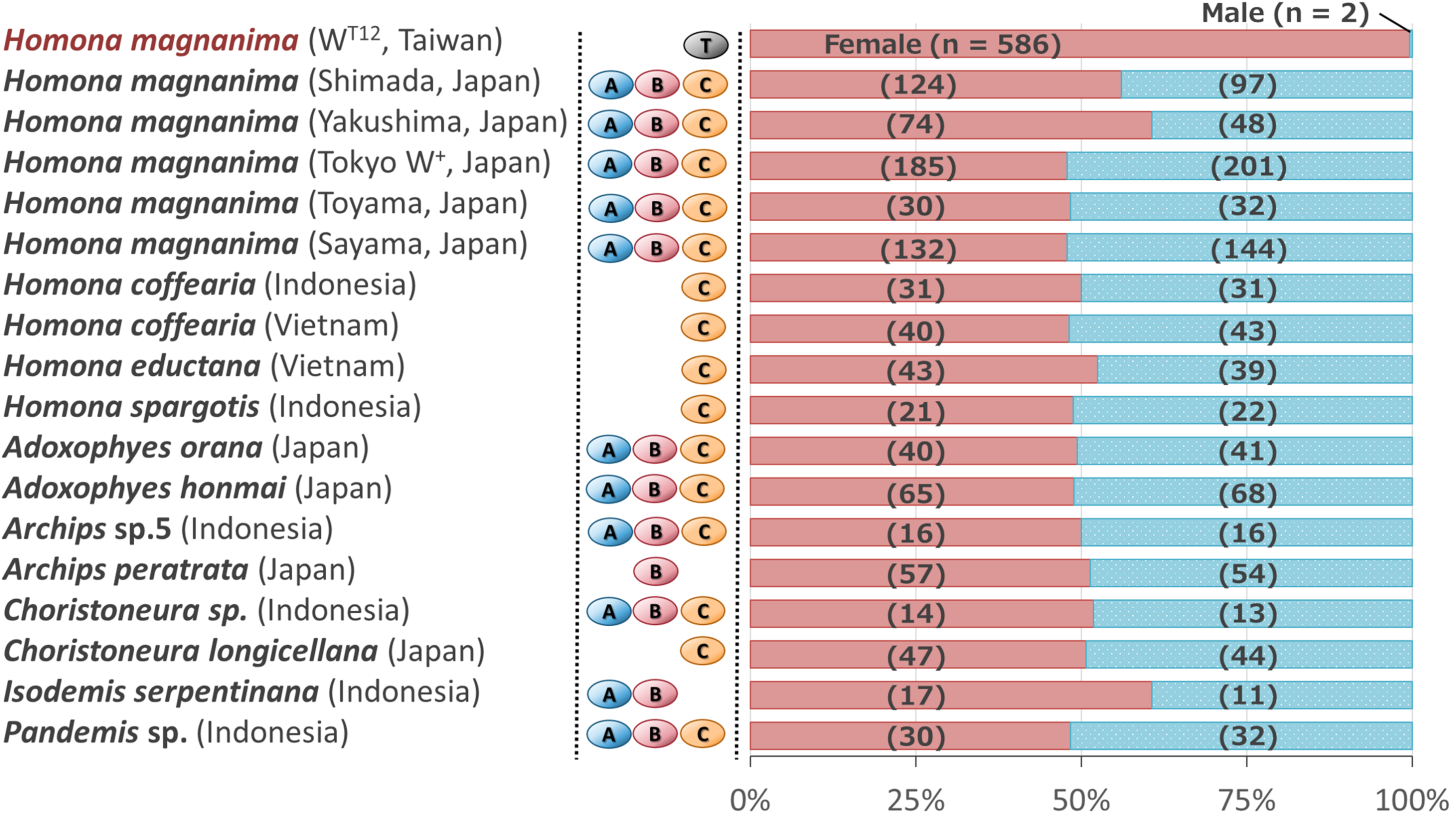
Sex ratios of tortricid species harbouring *Wolbachia* strains. *Wolbachia* infections are shown as A (*w*Hm‐a), B (*w*Hm‐b), C (*w*Hm‐c), and T (*w*Hm‐t). The sex ratio of each tortricid species is shown with red (female) and blue (male) bars. The numbers of female and male individuals are indicated within parentheses.

We collected more than 1300 tortricid samples, which were classified based on morphological characters described by Kishida (2011) and mitochondrial COI sequences, if available, in the BOLD database. In addition to *H. magnanima* (991 individuals), 61 tortricid species (357 individuals) were identified, although we were not able to clarify the species' name in some samples (e.g., *Archips* sp. 1, Table S1). Among them, 58 species were positive for *Wolbachia* (with 50–100% frequency, Table S1). Of these, 48, 45 and 54 were positive for *w*Hm‐a, *w*Hm‐b and *w*Hm‐c, respectively (Table S1). Similar to *H. magnanima* (Takamatsu et al., 2021), triple infections predominated (33 species), followed by dual infections with *w*Hm‐a and *w*Hm‐c (16 species), *w*Hm‐a and *w*Hm‐b (4 species) and *w*Hm‐b and *w*Hm‐c (8 species). Although no apparent topological concordance was observed between the *Wolbachia wsp* phylogeny and the tortricid *COI* phylogeny, many of the *Wolbachia* strains in tortricids shared highly homologous *wsp* sequences with those of either *w*Hm‐a, *w*Hm‐b or *w*Hm‐c in *H. magnanima* (Figure 1). The genus *Homona* typically harboured *w*Hm‐c but frequently lost *w*Hm‐a and *w*Hm‐b strains (Table S1; Figure 1). *Homona coffearia* and *Homona issikii* were co‐infected with *w*Hm‐c and other *Wolbachia* strains that were not identified from *H. magnanima*. Notably, males of 44 tortricid species harboured *w*Hm‐c (Table S1), and nine tortricid species harbouring *w*Hm‐c type *Wolbachia* did not exhibit a female‐biased sex ratio (Figure 2). To clarify whether the tortricid species harbour *w*Hm‐t, we detected the *Hm‐oscar* gene in the MK‐associated prophage WO*w*Hm‐t76 region that is present in *w*Hm‐t but absent in *w*Hm‐c (Arai et al., 2023). Apart from the Taiwanese *H. magnanima*, no tortricid was positive for the *Hm‐oscar* gene (Table S1).

### *Distinct* Wolbachia *infection status among populations and between* H. magnanima *sexes*

**FIGURE 3:**
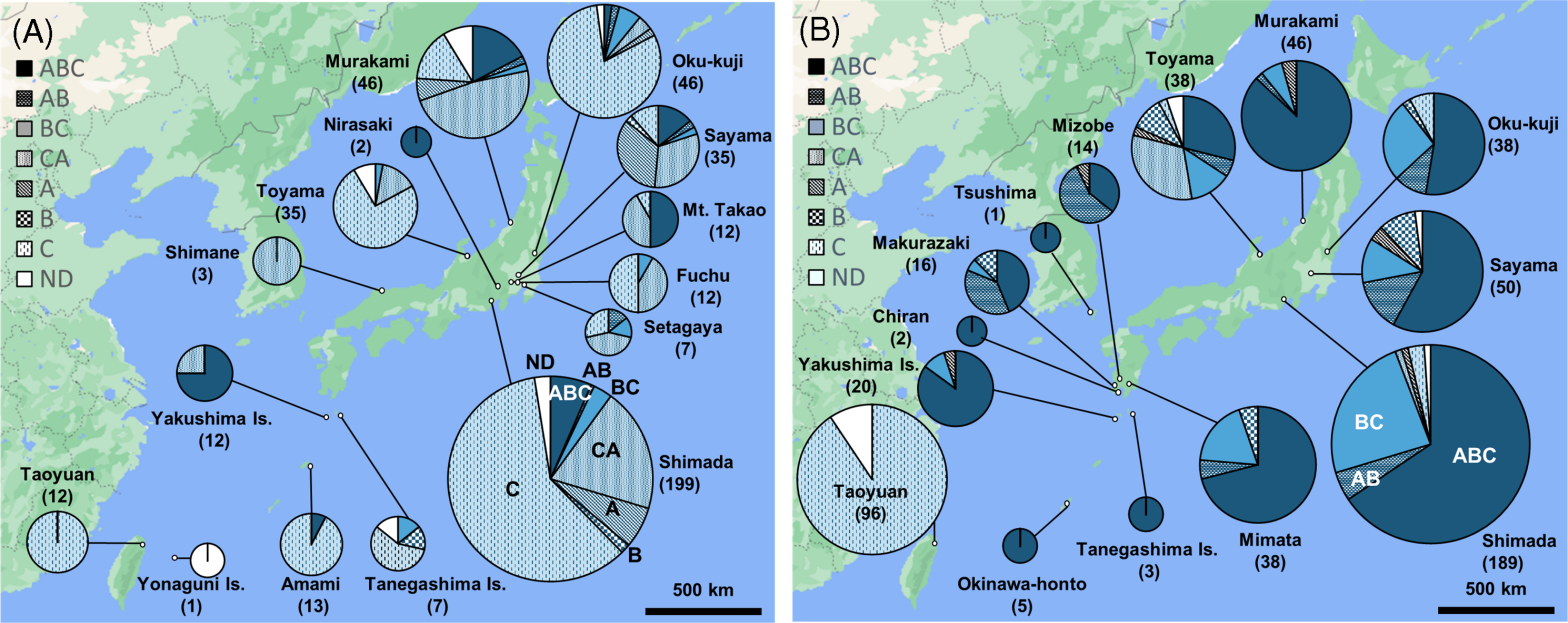
Distributions and infection status of *Wolbachia* strains in Japanese and Taiwanese *H. magnanima*. Pie charts of *Wolbachia* prevalence in males (A) and females (B). Taiwanese *w*Hm‐t strain is shown as a *w*Hm‐c type strain. ABC: *w*Hm‐a, *w*Hm‐b, and *w*Hm‐c triple infection; AB: *w*Hm‐a and *w*Hm‐b dual infection; BC: *w*Hm‐b and *w*Hm‐c dual infection; CA: *w*Hm‐a and *w*Hm‐c dual infection; A: *w*Hm‐a single infection; B: *w*Hm‐b single infection; C: *w*Hm‐c single infection; ND: not detected. *Wolbachia* prevalence data in Shimada and Taiwan were obtained from Takamatsu et al. (2021) and Arai et al. (2020), respectively.

Taiwanese *H. magnanima* harboured only the *w*Hm‐c relatives (i.e., *w*Hm‐t), whereas most Japanese populations harboured *w*Hm‐a, *w*Hm‐b and *w*Hm‐c (Figure 3, Table S1). Strikingly, Japanese *H. magnanima* males and females showed distinct infection patterns for *w*Hm‐a (generalised linear mixed model [GLMM], *p* < 0.001) and *w*Hm‐b (*p* < 0.001) but not *w*Hm‐c (*p* = 0.44), wherein *w*Hm‐a, *w*Hm‐b and *w*Hm‐c were 40.3%, 16.0% and 87.5% for males (*n* = 424; Figure 3A), and 76.7%, 91.9% and 85.4% for females, respectively (*n* = 459; Figure 3B). Triple infection was predominant in females (*n* = 290, 63.2% of the total individuals), whereas *w*Hm‐c single infection was predominant in males (218, 51.1%).

### *Taiwanese and Japanese* H. magnanima *harboured distinct mitochondrial haplotypes*

**FIGURE 4:**
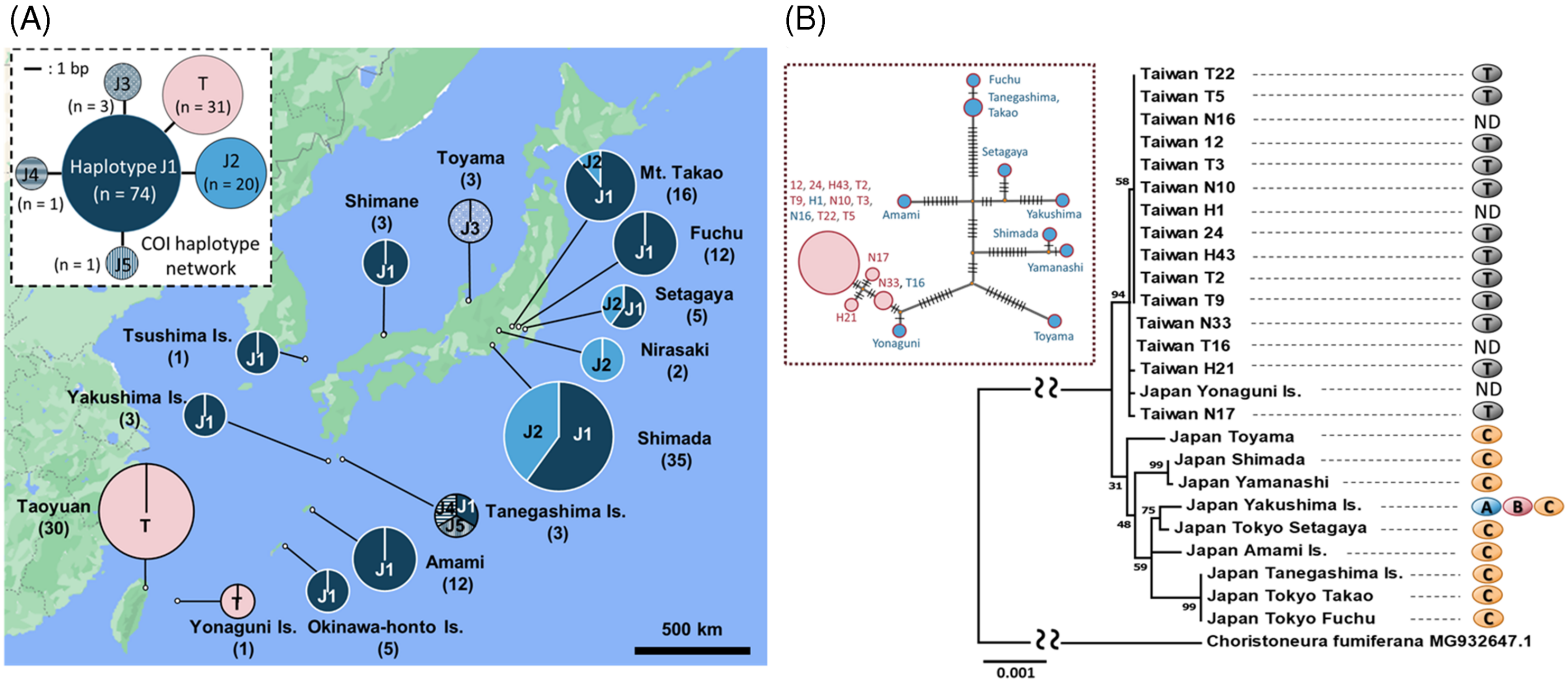
The mitochondrial haplotypes of *Homona magnanima*. (A) Distributions of the six *H. magnanima* mitochondrial *COI* haplotypes. Navy: haplotype J1; blue: haplotype J2; Navy with dots: haplotype J3; Navy with the horizontal line: haplotype J4; Navy with the diagonal line: haplotype J5; Red: haplotype T. Numbers of individuals are shown in parentheses. The *H. magnanima COI* haplotype network is highlighted by broken lines. (B) Haplotype network and phylogeny of *H. magnanima* mitochondria genome (16,529 bp). Red circle: Taiwanese individuals; blue circle: Japanese individuals; red letter: MK line; blue letter: non‐MK line or male individual. The numbers of Mutated positions are shown as black bars. A phylogenetic tree was constructed using the maximum likelihood method (based on the Hasegawa–Kishino–Yano model) with 1000 bootstrap replicates. A: *w*Hm‐a, B: *w*Hm‐b, C: *w*Hm‐c; T: *w*Hm‐t; ND: undetected.

Among 130 wild‐caught individuals of *H. magnanima*, the mitochondrial *COI* (599 bp) was polymorphic at six nucleotide sites, forming six haplotypes (Figure 4A). Taiwanese *H. magnanima* (*n* = 30) and a male individual collected from Yonaguni Island, which is closer to Taiwan (111 km away) than to Okinawa Island (509 km away), had the haplotype T. Of the remaining five haplotypes, haplotype J1 (*n* = 71) dominated most of the Japanese regions (mainland to Okinawa Island). Both haplotypes J1 and J2 were identified in the Tokyo and Shizuoka populations. Minor mitochondrial haplotypes (J3 to J5) were identified in Toyama (haplotype J3, n = 3) and Tanegashima (haplotype J4, *n* = 1; haplotype J5, *n* = 1). Furthermore, phylogenetic analysis using de novo assembled *H. magnanima* mitochondrial genomes (16,529 bp in length) confirmed more apparent divergent patterns among populations, divided into two distinct clades (Figure 4B). In addition, MK matrilines (*n* = 12) and non‐MK matrilines (*n* = 9) established from a Taiwanese *H. magnanima* population showed no apparent genetic divergence in mitochondrial sequences (Figure 4B).

## DISCUSSION

### *Evolutionary processes by which* Wolbachia *achieved MK in* H. magnanima

**FIGURE 5:**
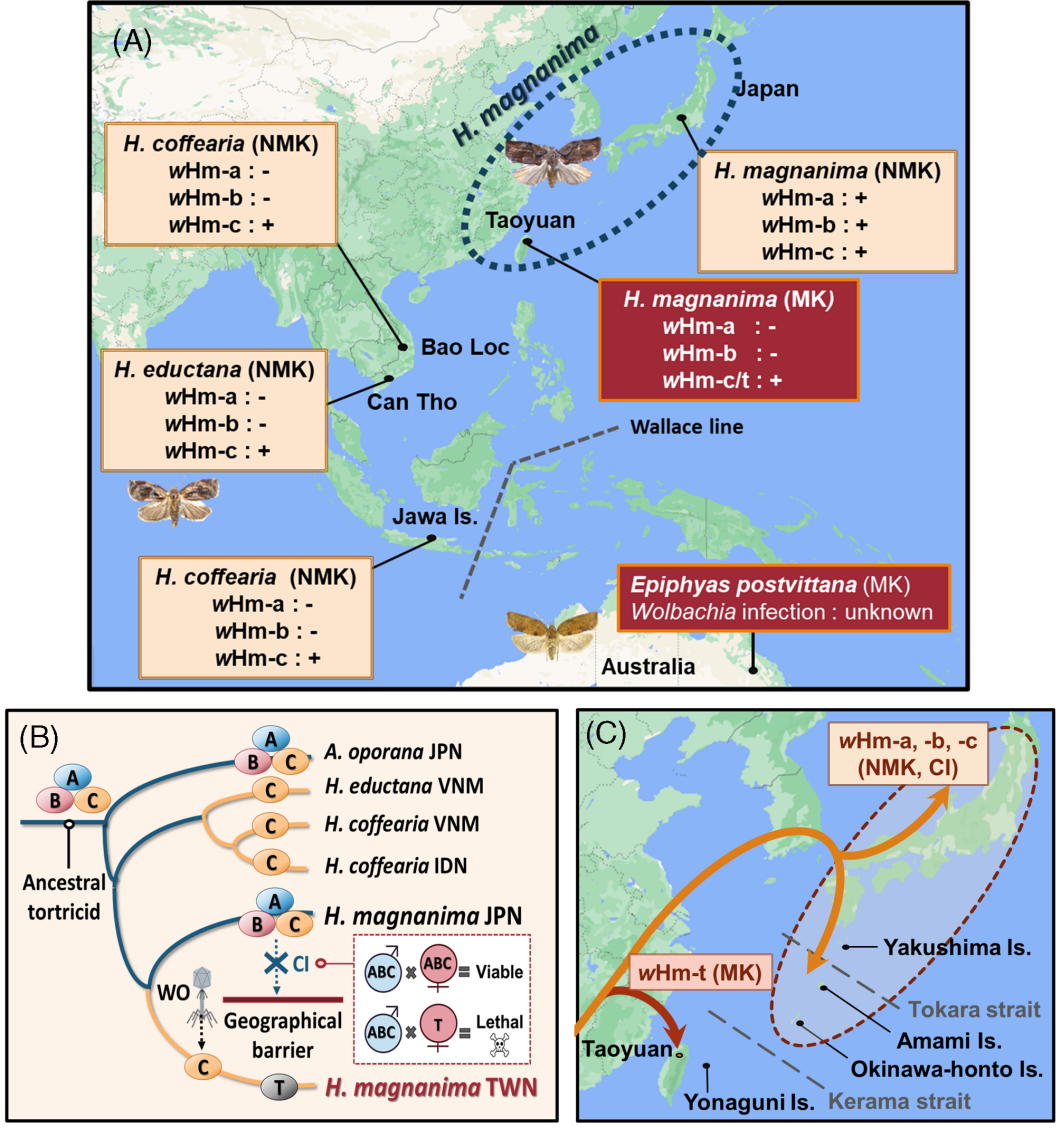
Distribution and male killing (MK) acquisition scenarios of *Wolbachia* in Tortricidae. (A) Overview of *Wolbachia* infections, distributions, and MK in the *Homona* species examined in this study. The Wallace line (broken grey line) is a border of organisms between Asian and Australian regions. (B) Evolutionary scenarios of the MK *w*Hm‐t. While *Wolbachia* strains have descended from the ancestral host, *w*Hm‐t has evolved from *w*Hm‐c through phage infection in *H. magnanima*. Cytoplasmic incompatibility (CI) may have limited the distribution of *w*Hm‐t infected hosts, as crosses between *w*Hm‐t infected females (shown as T) and triply‐infected males (shown as ABC) are lethal. JPN: Japan; VNM: Vietnam; IDN: Indonesia; TWN: Taiwan. (C) A putative evolutionary process of the Japanese and Taiwanese *H. magnanima*. Ancient *H. magnanima* diverged in mainland China, and triple‐infected *H. magnanima* that entered the Japanese archipelago via the Korea–Tsushima route (shown as orange arrows) formed the Japanese population. *H. magnanima* infected with *w*Hm‐t was not expelled in Taiwan (Taoyuan).

Here we showed that *Wolbachia* relatives of *w*Hm‐a, *w*Hm‐b and *w*Hm‐c were conserved among Asian tortricids, suggesting that these strains descended from a common tortricid ancestor. We also confirmed that *w*Hm‐c relatives in tortricid species were negative for the *w*Hm‐t‐specific prophage gene and did not induce MK. Apart from *H. magnanima*, MK in tortricids has only been reported in a severe pear pest *Epiphyas postvittana*, which is distributed in Australasia (Geier et al., 1978, Figure 5A). Although MK factors in *E. postvittana* are currently unknown, the different distributions of *H. magnanima* (East Asia) and *E. postvittana* (Australia) suggest that they have acquired MK independently. In *H. magnanima*, Japanese and Taiwanese populations had different mitochondrial genotypes. Furthermore, there was no genetic divergence between the MK and non‐MK Taiwanese host lines. These results suggest that the MK trait of *w*Hm‐t has emerged relatively recently, at least after the Taiwanese *H. magnanima* had diverged from the Japanese populations. The triply infected *H. magnanima* (ancestor of the Taiwanese population) had probably lost *w*Hm‐a and *w*Hm‐b, as is frequently observed in other species of the genus *Homona*. Thereafter, we suspect that the ancestral *w*Hm‐c type *Wolbachia* in *H. magnanima* acquired an MK ability (i.e., the MK‐associated prophage region WO*w*Hm‐t76; Arai et al., 2023) through a bacteriophage integration, giving rise to the MK‐inducing *w*Hm‐t (Figure 5B).

### *Sex‐linked infection patterns of non‐MK* Wolbachia *in* H. magnanima

We found that *w*Hm‐a and *w*Hm‐b show distinct infection patterns between male and female *H. magnanima*. Both strains possess the CI causative genes *cifA* and *cifB* (Arai et al., 2023), although *w*Hm‐a does not currently induce CI in contrast to *w*Hm‐b (Arai et al., 2019). Similar differences in *Wolbachia* infection patterns between the sexes have been reported in several insects, such as Diptera (*Drosophila pseudotakahashii* and *Aedes albopictus*), Coleoptera (*Polygraphus proximus*) and Siphonaptera (*Synosternus cleopatrae*) (Bykov et al., 2020; Cohen et al., 2015; Dobson et al., 2002; Dutton & Sinkins, 2004; Flatau et al., 2018; Richardson et al., 2019; Xi et al., 2005; Xi et al., 2006). For example, females of *A. albopictus* carry CI‐inducing *Wolbachia* strains *w*AlbA and *w*AlbB, but males often lack *w*AlbA or carry it at very low densities (Dobson et al., 2002; Dutton & Sinkins, 2004; Xi et al., 2005; Xi et al., 2006). Although the evolutionary history of the distinct *Wolbachia* infection patterns between males and females remains unknown, it may reflect a host response to *Wolbachia*‐induced reproductive manipulations such as CI. CI‐inducing *Wolbachia* causes lethality in offspring when infected males mate with uninfected females (Werren et al., 2008). Furthermore, as observed in the *A. albopictus*‐*Wolbachia* (*w*AlbA, *w*AlbB and *w*Mel) system, multiple infections of CI‐inducing *Wolbachia* can reduce host fitness by leading to self‐incompatibility (Ant & Sinkins, 2018). Theory suggests that mutation(s) can evolve to suppress the proliferation and/or functions of CI‐inducing *Wolbachia* (Koehncke et al., 2009). If CI‐inducing *Wolbachia* cannot be eliminated from the host, low frequencies of CI‐inducing *Wolbachia* in males and high frequencies in females are assumed to be adaptive for host insects to produce viable offspring. We speculate that *H. magnanima* has developed mechanisms to control CI‐inducing *Wolbachia* strains through evolutionary processes. Although the underlying mechanisms are unknown, we hypothesise that differences in the male and female environments (e.g., metabolites and/or tissues) regulate *Wolbachia–Wolbachia* interactions and their proliferation, resulting in distinct infection patterns between the sexes.

Alternatively, this phenomenon may be a strategy of *Wolbachia* rather than the host. It is possible that *Wolbachia* adopted a strategy of allocating resources to females rather than males. In *D. pseudotakahashii*, *Wolbachia* expresses strong CI (a long‐lasting sperm modification effect) at extremely low titers in adult males (Richardson et al., 2019). The different infection patterns of *Wolbachia* between the sexes may be an evolutionarily acquired strategy of *Wolbachia*—low levels of *Wolbachia* being allocated to males only to induce CI, while high levels of *Wolbachia* to females to secure a stable infection. In *H. magnanima*, *w*Hm‐a and *w*Hm‐b may have systems to transmit to female siblings rather than males by recognising female‐specific features such as the W chromosome. Further investigation is warranted to elucidate the underlying mechanisms and whether this phenomenon occurs in other host *Wolbachia* systems.

### *Formation of the current distribution of* Wolbachia *and* H. magnanima

*Wolbachia* impacts genetic differentiation and distribution in insects (Kondo et al., 2005; Narita et al., 2007; Miyata et al., 2017; Miyata et al., 2020; Rokas, 2000; Turelli & Hoffmann, 1991). It is known that CI traits have led to the rapid spread of *Wolbachia* in *Drosophila simulans*, reducing the diversity of mitochondrial haplotypes in infected populations (Hale & Hoffmann, 1990; Ballad et al., 1996). In this study, we showed that a mitochondrial *COI* haplotype and three *Wolbachia* strains (*w*Hm‐a, *w*Hm‐b and *w*Hm‐c) were highly conserved among Japanese *H. magnanima*. We speculate that the CI‐inducing *w*Hm‐b led to the spread of the triply infected *H. magnanima* and its mitochondria haplotype across Japan, followed by the emergence of the minor haplotypes in Japanese *H. magnanima*. In contrast to most Japanese populations, Taiwanese *H. magnanima* does not harbour *w*Hm‐a or *w*Hm‐b and has a homogeneous mitochondrial sequence. Notably, a *Wolbachia*‐free male collected from Yonaguni Island had the Taiwanese mitochondrial haplotype. These findings suggest that the Taiwan–Yonaguni and mainland–Okinawa Island populations of *H. magnanima* have different origins and have been isolated over a long period. We speculate that the triply infected *H. magnanima* spread from mainland Japan across the Tokara Strait—a geographical boundary located between Yakushima Island and Amami‐Oshima Island (Tojo et al., 2017)—to Okinawa Island. The Kerama Strait (Hachisuka Line, Figure 5C) has probably served as the geographical boundary between the *H. magnanima* populations of Taiwan–Yonaguni and mainland–Okinawa Island. Given that *w*Hm‐t does not confer any fitness advantages to *H. magnanima* (Arai et al., 2020), the *w*Hm‐t infected host struggles to coexist with the triply infected host that retains the fitness advantages of *w*Hm‐c and the CI traits of *w*Hm‐b (Arai et al., 2019). Therefore, *w*Hm‐t infected hosts are likely to be eliminated by competition from triply infected hosts when they coexist in the same population. We propose that the triple infection of *w*Hm‐a, *w*Hm‐b and *w*Hm‐c have determined the limited distribution of the MK strain *w*Hm‐t.

## CONCLUSIONS

Our study demonstrated that *Wolbachia* relatives of *w*Hm‐a, *w*Hm‐b and *w*Hm‐c are widely conserved among Asian tortricids but do not induce MK in their hosts. Furthermore, Japanese and Taiwanese *H. magnanima* exhibit genetic divergence and distinct *Wolbachia* infection patterns. These results suggest that the *Wolbachia* strains are derived from a common ancestor, whereas the MK *w*Hm‐t has evolved from a non‐MK *w*Hm‐c strain in the ancestral Taiwanese *H. magnanima* that lacked *w*Hm‐a and *w*Hm‐b infections. This work is relevant in that we presented the evolutionary process of *Wolbachia* by examining 62 tortricid species collected from 39 localities across East and Southeast Asia. However, we must note that the phylogeny and infection dynamics of *Wolbachia* may be more complicated because the genus Tortricidae is a large taxon with more than 10,000 species in diverse environments around the world (http://www.tortricidae.com/catalogue.asp). Further studies on *Wolbachia* in tortrix moths and other insects should clarify the dynamics of the acquisition process of the MK phenotype.

## AUTHOR CONTRIBUTIONS

**Hiroshi Arai:** Conceptualization (lead); data curation (lead); formal analysis (lead); funding acquisition (equal); investigation (lead); methodology (lead); project administration (equal); resources (equal); supervision (equal); validation (equal); visualization (equal); writing – original draft (equal); writing – review and editing (equal). **Masatoshi Ueda:** Data curation (supporting); investigation (supporting); methodology (supporting); resources (supporting); validation (supporting); writing – review and editing (supporting). **Tatsuya Hirano:** Investigation (supporting); methodology (supporting); resources (supporting); writing – review and editing (supporting). **Naoya Akizuki:** Investigation (supporting); writing – review and editing (supporting). **Shiou‐Ruei Lin:** Conceptualization (supporting); investigation (equal); project administration (supporting); resources (equal); supervision (supporting); validation (supporting); writing – original draft (supporting); writing – review and editing (equal). **Duong Kieu Hanh:** Investigation (supporting); methodology (supporting); project administration (supporting); resources (supporting); writing – review and editing (supporting). **Jaka Widada:** Investigation (supporting); project administration (supporting); supervision (supporting); writing – review and editing (supporting). **Muhammad Saifur Rohman:** Investigation (supporting); methodology (supporting); project administration (supporting); supervision (supporting); writing – review and editing (supporting). **Madoka Nakai:** Funding acquisition (supporting); investigation (supporting); writing – review and editing (supporting). **Yasuhisa Kunimi:** Conceptualization (supporting); data curation (supporting); investigation (supporting); project administration (supporting); resources (supporting); supervision (supporting); validation (supporting); writing – review and editing (supporting). **Le Van Vang:** Conceptualization (equal); investigation (supporting); project administration (supporting); resources (equal); supervision (equal); writing – review and editing (supporting). **Arman Wijonarko:** Conceptualization (supporting); funding acquisition (supporting); investigation (supporting); methodology (supporting); project administration (supporting); resources (equal); supervision (equal); writing – review and editing (equal). **Maki Inoue:** Conceptualization (equal); funding acquisition (equal); project administration (lead); supervision (lead); writing – review and editing (equal).

## CONFLICT OF INTEREST STATEMENT

The authors declare that they have no conflicts of interest.

## Supporting information


**TABLE S1:** Wolbachia infection status of the field‐collected tortricids.

## Data Availability

The sequence data were deposited in GenBank under accession numbers LC741220 to LC741253 and LC741449. The *Homona magnanima* were collected from Tea Research and Extension Station (Taoyuan City, Taiwan) and were imported with permission from the Ministry of Agriculture, Forestry and Fisheries (No. 27—Yokohama Shokubou 891 and No. 297—Yokohama Shokubou 1326). A research collaboration was developed with scientists from the countries providing genetic samples; all collaborators are included as co‐authors, and the research results have been shared with the provider communities and the broader scientific community. Our group is committed to international scientific partnerships and institutional capacity building.
